# Early prognostic stratification and identification of irreversibly shocked patients despite primary percutaneous coronary intervention

**DOI:** 10.2459/JCM.0000000000001282

**Published:** 2021-12-13

**Authors:** Luca Falco, Enrico Fabris, Caterina Gregorio, Andrea Pezzato, Marco Milo, Laura Massa, Gerardina Lardieri, Renata Korcova, Franco Cominotto, Giancarlo Vitrella, Serena Rakar, Andrea Perkan, Gianfranco Sinagra

**Affiliations:** a Cardiothoracovascular Department; b Biostatistics Unit, Department of Medical Sciences, University of Trieste, Trieste; c Division of Cardiology, Emergency Department, Gorizia–Monfalcone Hospital; d Emergency Department, University Hospital of Trieste, Trieste, Italy

**Keywords:** cardiogenic shock, elderly, myocardial infarction, prehospital stratification, primary percutaneous coronary intervention

## Abstract

**Background:**

Despite prognostic improvements in ST-elevation myocardial infarction (STEMI), patients presenting with cardiogenic shock (CS) have still high mortality. Which are the relevant early prognostic factors despite revascularization in this high-risk population is poorly investigated.

**Methods:**

We analyzed STEMI patients treated with primary percutaneous coronary intervention (PCI) and enrolled at the University Hospital of Trieste between 2012 and 2018. A decision tree based on data available at first medical contact (FMC) was built to stratify patients for 30-day mortality. Multivariate analysis was used to explore independent factors associated with 30-day mortality.

**Results:**

Among 1222 STEMI patients consecutively enrolled, 7.5% presented with CS. CS compared with no-CS patients had worse 30-day mortality (33% vs 3%, *P* < 0.01). Considering data available at FMC, CS patients with a combination of age ≥76 years, anterior STEMI and an expected ischemia time > 3 h and 21 min were at the highest mortality risk, with a 30-day mortality of 85.7%. In CS, age (OR 1.246; 95% CI 1.045–1,141; *P* = 0.003), final TIMI flow 2–3 (OR 0.058; 95% CI 0.004–0.785; *P* = 0.032) and Ischemia Time (OR = 1.269; 95% CI 1.001–1.609; *P* = 0.049) were independently associated with 30-day mortality.

**Conclusions:**

In a contemporary real-world population presenting with CS due to STEMI, age is a relevant negative factor whereas an early and successful PCI is positively correlated with survival. However, a subgroup of elderly patients had severe prognosis despite revascularization. Whether pPCI may have an impact on survival in a very limited number of irreversibly critically ill patients remains uncertain and the identification of irreversibly shocked patients remains nowadays challenging.

## Introduction

ST-segment elevation myocardial infarction (STEMI) incidence in Europe ranges from 43 to 144 per 100,000 per year^1^ and despite modern STEMI network organization, reperfusion techniques and new antithrombotic therapies^2^ is still burdened by significant mortality.^3^

Primary percutaneous coronary intervention (pPCI) is the treatment of choice in STEMI patients, and it must be performed as soon as possible in order to improve complication rates and outcomes.^4,5^

Several conditions are known to be associated with increased mortality: age, Killip class, time delay to treatment, treatment strategy, previous myocardial infarction, diabetes mellitus, etc.^6–10^ Among these, cardiogenic shock (CS) presentation, defined as persistent hypotension despite adequate filling status with signs of hypoperfusion, remains a leading cause of death, with in-hospital mortality rates up to 50% and over.^11^ Moreover, the combination of these factors may exert an exponentially detrimental effect on survival, making pPCI futile in some irreversibly critically ill patients.

Some of the key points of the management of STEMI reported by guidelines are based on randomized trials.^7,12,13^ However, one of the major limitations of clinical trials performed in the emergency setting is their applicability in the real world. Indeed real-world registry data may evaluate some specific populations which are often excluded by randomized trials, due to the complexity of the clinical scenario and also difficulties in obtaining informed consent, as in patients presenting with CS. It is important to understand challenges in clinical practice, so the aim of our study was to evaluate our registry data in order to understand the characteristics of the CS population, the outcomes, and to identify those patients who, based on data available at the first medical contact (FMC), have the highest mortality risk.

## Methods

### Population and outcome

We analyzed all the STEMI patients undergoing pPCI, consecutively enrolled in the Trieste University Hospital pPCI registry from January 2012 to June 2018, in order to have a homogeneous population regarding technical strategies and pharmacological treatment. Acute STEMI was defined as:

(a) presentation within 12 h of typical symptoms onset; (b) 1 mm or greater ST-segment elevation in two contiguous leads of electrocardiogram (ECG) (≥2 mm in precordial leads).

CS was defined as:

(a) persistent hypotension [systolic blood pressure (SBP) below 90 mmHg, or need for inotropes and/or vasopressor in order to maintain SBP over 90 mmHg];

(b) evidence of end-organ hypoperfusion (cold/clammy extremities, oliguria, altered mental status, high lactate levels: >2.0 mmol per liter).

The main outcomes investigated in this study were in the hospital, 30 days and follow-up all-cause mortality in the CS population. A ‘decision tree’ based on the sole data available at the moment of FMC of a patient with STEMI who was a candidate for pPCI (CS, age, STEMI localization and ischemia time) was built to identify the population at the highest mortality risk.

### Statistical analysis

Data on continuous scale are reported as mean and standard deviation or median and interquartile range, as appropriate; categorical variables are reported as counts and percentages. Descriptive comparisons between groups were tabulated and tested by means of ANOVA test on continuous variables, using the Brown–Forsythe robust tests when appropriate or the nonparametric Mann–Whitney test for non-Gaussian variables. The Chi-square test or Fisher exact test was calculated on categorical parameters. To identify factors independently associated with the selected outcomes in the CS population, a multivariate logistic regression model using forward stepwise selection was performed. Kaplan–Meier survival curves were estimated and compared between groups using a log-rank test. Finally, we designed a decision tree based on the parameters readily available at the moment of the FMC: CS, infarction site, age and total ischemia time. Briefly, decision tree methodology is a commonly used data mining method for establishing classification systems based on multiple covariates or for developing prediction algorithms for a target variable.^14^ The decision tree was created using the Quick, Unbiased and Efficient Statistical Tree analysis, a tool that uses ANOVA and contingency table Chi-square tests to select variables for splitting and applies quadratic discriminant analysis to determine the split point. IBM SPSS software was used for the analysis. The datasets generated and/or analyzed during the current study are not publicly available.

## Results

From 1 January 2012 to 30 June 2018, 1222 consecutive patients diagnosed with STEMI were treated with pPCI. The most relevant risk factors were hypertension (50.5%) and smoke (46.3%), whereas 15.7% of patients had diabetes mellitus; 21% of patients had chronic kidney disease (CKD, defined as eGFR <60 mL/min/m^2^, calculated with Modification of Diet in Renal Disease formula).

Anterior STEMI were 41%, 42.6% had multivessel coronary disease at index angiography and 10.5% of STEMIs were complicated by cardiac arrest. Complete characteristics of the overall population are listed in Table 1. Among these patients, 91 (7.5%) presented with CS. Descriptive characteristics of the population according to CS status are presented in Table 2.

**Table 1:** Baseline characteristics

Patient characteristics	
Age (years)	65 ± 12
Age ≥75 (%)	330 (27)
Males (%)	901 (74)
Past medical history	
Previous MI (%)	107 (8.8)
Previous PCI (%)	102 (8.3)
Hypertension (%)	617 (50.5)
Diabetes mellitus (%)	192 (15.7)
Family history of CAD (%)	298 (24.4)
Smoke (%)	566 (46.3)
Dyslipidemia (%)	565 (46.2)
Peripheral artery disease (%)	49 (4.0)
Clinical variables	
Heart rate (bpm)	75 ± 18
Systolic blood pressure (mmHg)	132 ± 30
Diastolic blood pressure (mmHg)	74 ± 16
LVEF (at admission) [in %]	49 ± 11
LVEF <40% at admission (%)	153 (16.2)
Anterior MI (%)	502 (41)
Right ventricular MI (%)	58 (4.7)
Cardiac arrest (%)	129 (10.5)
OTI	71 (5.8)
Laboratory findings	
Glycemia mg/dL	163 ± 67
Creatinine mg/dL	1.01 ± 0.56
eGFR <60 mL/min/m^2^ (%)	174 (21.3)
Hemoglobin g/dL	13.5 ± 2.0
Ischemia time (h:min, IQR)	3:09 (2:13–5:15)
Angiographic variables	
Multivessel disease (%)	521 (42.6%)
Initial TIMI flow 2–3 (%)	307 (25.1)
Final TIMI flow 2–3 (%)	1157 (94.7)
Procedural variables	
Stent implanted	1.24 ± 0.66
IABP (%)	52 (4.2)
ECMO	3 (0.2)
GPIIb/IIIa inhibitors (%)	266 (21.8)
Post-PCI variables	
Peak troponin I (ng/mL)	110 ± 113
Q wave (%)	655 (68)
LVEF at discharge (%)	47 ± 17
Pharmacological therapy	
Aspirin (%)	927 (95.9)
P2Y12-inhibitors	922 (95.4)
Clopidogrel (%)	313 (33.9)
Prasugrel (%)	510 (55.3)
Ticagrelor (%)	99 (10.7)
Beta blocker (%)	792 (82.7)
ACE inhibitor (%)	722 (75.2)
Statin (%)	911 (94.9)
Outcomes	
In-hospital mortality (%)	62 (5.1)
30-day mortality (%)	65 (5.4)
Long-term mortality^a^	132 (10.8)

a Median follow-up 38 (IQR 16–57) months. CAD, coronary artery disease; GP, glycoprotein; LVEF, left ventricular ejection fraction; MI, myocardial infarction; OTI, orotracheal intubation.

**Table 2:** Descriptive analysis in patients with and without cardiogenic shock

	Shock	No shock	*P*-value
	(*N* = 91)	(*N* = 1131)	
Patient characteristics			
Age (years)	71 ± 12	65 ± 12	<0.001
Age ≥75 (%)	42 (46.2)	288 (25.5)	<0.001
Males (%)	56 (62.2)	845 (74.9)	0.012
Past medical history			
Previous MI (%)	4 (5)	103 (10.9)	0.125
Previous PCI (%)	3 (3.8)	99 (10.4)	0.52
Hypertension (%)	50 (62.5)	567 (59.9)	0.722
Diabetes mellitus (%)	19 (23.8)	173 (18.3)	0.233
Family history of CAD (%)	16 (20)	282 (29.9)	0.072
Smoke (%)	38 (47.5)	528 (55.8)	0.161
Dyslipidemia (%)	39 (48.8)	526 (55.6)	0.244
Peripheral artery disease (%)	7 (8.8)	42 (4.4)	0.096
Clinical variables			
Heart rate (bpm)	74 ± 32	76 ± 17	0.762
Systolic blood pressure (mmHg)	90 ± 25	136 ± 28	<0.001
Diastolic blood pressure (mmHg)	52 ± 18	76 ± 15	<0.001
LVEF (at admission)	41 ± 12	50 ± 10	<0.001
LVEF <40% at admission (%)	25 (49)	128 (14,3)	<0.001
Anterior MI (%)	44 (49.4)	459 (43.2)	0.267
Right ventricular MI (%)	23 (25.8)	35 (3.3)	<0.001
Cardiac arrest (%)	27 (29.7)	102 (9)	<0.001
OTI	27 (29.7)	44 (3.9)	<0.001
Laboratory findings			
Glycemia mg/dL	222 ± 93	158 ± 62	<0.001
Creatinine mg/dL	1.34 ± 1.03	0.87 ± 0.50	0.011
eGFR <60 mL/min/m^2^ (%)	29 (50)	164 (21.1)	<0.001
Hemoglobin g/dL	12.4 ± 2.9	13.6 ± 2	0.003
Ischemia time (h:min, IQR)	2:50 (2:03–5:09)	3:09 (2:14–5:16)	<0.001
Angiographic variables			
Multivessel disease (%)	51 (57.3)	470 (42)	0.005
Initial TIMI flow 2–3 (%)	12 (13.6)	295 (26.3)	0.007
Final TIMI flow 2–3 (%)	78 (87.6)	1079 (96.3)	0.001
Procedural variables			
Stent implanted	1.27 ± 0.83	1.24 ± 0.65	0.679
IABP (%)	27 (30.3)	25 (2.2)	<0.001
ECMO	3 (3.2%)	0	<0.001
GPIIb/IIIa inhibitors (%)	22 (24.7)	244 (21.7)	0.507
Post-PCI variables			
Peak troponin I ng/mL	157 ± 142	105 ± 109	0.025
Q wave (%)	46 (75.4)	609 (67.5)	0.256
LVEF at discharge	39 ± 20	48 ± 17	0.005
Pharmacological therapy			
Aspirin (%)	53 (96.4)	874 (95.8)	0.845
P2Y12-inhibitors	47 (87.0)	875 (95.8)	0.003
Clopidogrel (%)	22 (40.7)	291 (32.0)	0.182
Prasugrel (%)	16 (30.2)	494 (54.5)	0.001
Ticagrelor (%)	9 (22.5)	90 (14.8)	0.188
Beta blocker (%)	33 (62.3)	759 (83.9)	<0.001
ACE inhibitor (%)	34 (64.2)	688 (76.1)	0.039
Statin (%)	48 (90.6)	863 (95.3)	0.180
Outcomes			
In-hospital mortality (%)	29 (31.9)	33 (2.9)	<0,001
30-day mortality (%)	30 (33)	35 (3.1)	<0.001
Long-term mortality^a^	37 (40.7)	95 (8.4)	<0.001

a Median follow-up 38 (IQR 16–57) months. CAD, coronary artery disease; GP, glycoprotein; LVEF, left ventricular ejection fraction; MI, myocardial infarction; OTI, orotracheal intubation.

### Cardiogenic shock population compared to no cardiogenic shock

**Fig. 1:**
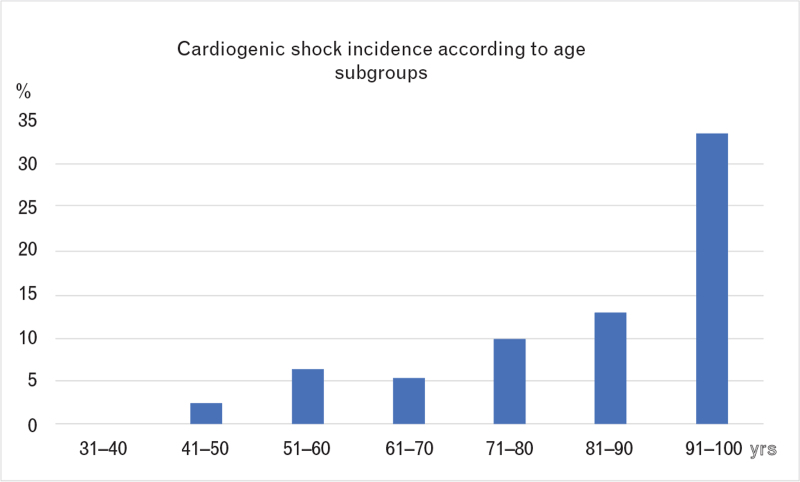
Percentage of cardiogenic shock according to age.

The CS population compared with the no-CS population had a lower percentage of males (62% vs 75%, *P* < 0.001) and was significantly older (71 vs 65 years old, *P* < 0.01). Moreover, the incidence of CS was higher in older patients (Fig. 1). CS patients compared with no-CS patients had more frequently cardiac arrest presentation (30% vs 9%, *P* < 0.001), worse renal function (CKD prevalence 50% vs 21%, *P* < 0.001), had a more severe coronary artery disease (multivessel disease in 57% vs 42%, *P* = 0.005) and worse final angiographic results (TIMI flow 2 or 3 in 88% vs 96%, *P* = 0.007). Overall ischemia time was borderline significantly shorter in CS patients (2 h 50 min vs 3 h 10 min, *P* = 0.079).

### Outcomes

In the overall STEMI population in-hospital mortality was 5.1%, 30-day mortality was 5.4% and 10.8% at a median follow-up of 38 (Inter Quartile Range 16–57) months.

The CS population compared with the no-CS population presented as severely higher in hospital mortality (32% vs 3%, *P* < 0.001), 30-day mortality (33% vs 3%, *P* < 0.001), and mortality at median follow-up of 38 (IQR 16–57) months (41% vs 8%, *P* < 0.001).

Descriptive analysis of patients with CS according to 30-day mortality is presented in Table 3.

**Table 3:** Descriptive analysis in patients with cardiogenic shock alive and dead 30 days after pPCI

	Alive	Dead	*P*-value
	(*N* = 61)	(*N* = 30)	
Patient characteristics			
Age (years)	69 ± 12	76 ± 10	0.003
Age ≥75 (%)	21 (33)	21 (67)	0.002
Males (%)	36 (60)	20 (67)	0.647
Past medical history			
Previous MI (%)	4 (7,7)	0 (0)	0.292
Previous PCI (%)	3 (5,8)	0 (0)	0.548
Hypertension (%)	35 (67)	15 (50)	0.238
Diabetes mellitus (%)	15 (29)	4 (14)	0.177
Family history of coronary artery disease (%)	15 (29)	1 (3.6)	0.007
Smoker (%)	25 (48)	13 (46)	0.538
Dyslipidemia (%)	26 (50)	13 (46)	0.244
Peripheral artery disease (%)	3 (5,8)	4 (14)	0.232
Clinical variables			
Heart rate (bpm)	69 ± 30	85 ± 25	0.048
Systolic blood pressure (mmHg)	92 ± 28	87 ± 18	0.339
Diastolic blood pressure (mmHg)	54 ± 18	49 ± 19	0.292
LVEF (at admission)	43 ± 10	36 ± 16	0.142
Anterior MI (%)	25 (42)	19 (63)	0.050
Right ventricular MI (%)	17 (29)	6 (20)	0.448
Cardiac arrest (%)	16 (26)	12 (37)	0.336
OTI	15 (24,6)	12 (40)	0.130
Laboratory findings			
Glycemia mg/dL	220 ± 94	227 ± 92	0.794
Creatinine mg/dL	1.14 ± 0.55	1.87 ± 1.67	0.014
eGFR <60 mL/min/m^2^ (%)	21 (46)	12 (75)	0.040
Hemoglobin g/dL	12.9 ± 1.8	11.4 ± 4,5	0.080
Ischemia time (h:min, IQR)	2:39 (1:51–4:27)	3:35 (2:17–5:15)	<0.001
Angiographic variables			
Multivessel disease (%)	29 (48)	22 (73)	0.041
Initial TIMI flow 2–3 (%)	7 (12)	5 (17)	0.534
Final TIMI flow 2–3 (%)	57 (97)	21 (70)	0.001
Procedural variables			
Stent implanted	1.3 ± 0.69	1.2 ± 1.10	0.593
IABP (%)	16 (27)	11 (37)	0.465
ECMO	2 (3.3)	1 (3,3)	0.989
GPIIb/IIIa inhibitor (%)	18 (30)	4 (13)	0.118
Catecholamines	35 (57.4)	30 (100)	<0.001
Adrenaline	4 (6.6)	12 (40.0)	<0.001
Norepinephrine	18 (29.5)	14 (46.7)	0.107
Dobutamine	14 (23.0)	11 (36.7)	0.168
Dopamine	11 (18.0)	6 (20.0)	0.821
Mechanical complications	2 (3.3)	2 (6.7)	0.596

CAD, coronary artery disease; GP, glycoprotein; LVEF, left ventricular ejection fraction; MI, myocardial infarction; OTI, orotracheal intubation.

Alive patients at 30 days compared with dead patients were younger (69 vs 76 years old, *P* = 0.003), had lower heart rate (69 vs 85 bpm, *P* = 0.048), had less frequently an anterior STEMI (42% vs 63%, *P* = 0.050), had less frequently CKD (46% vs 75%, *P* = 0.040), had less frequently a multivessel disease (49% vs 73%, *P* = 0.041) and more frequently a final TIMI flow 2 or 3 (97% vs 70%, *P* = 0.001). Finally, ischemia time was significantly shorter among patients alive at 30 days (2:39 h:min vs 3:35 h:min, *P* < 0.001).

**Fig. 2:**
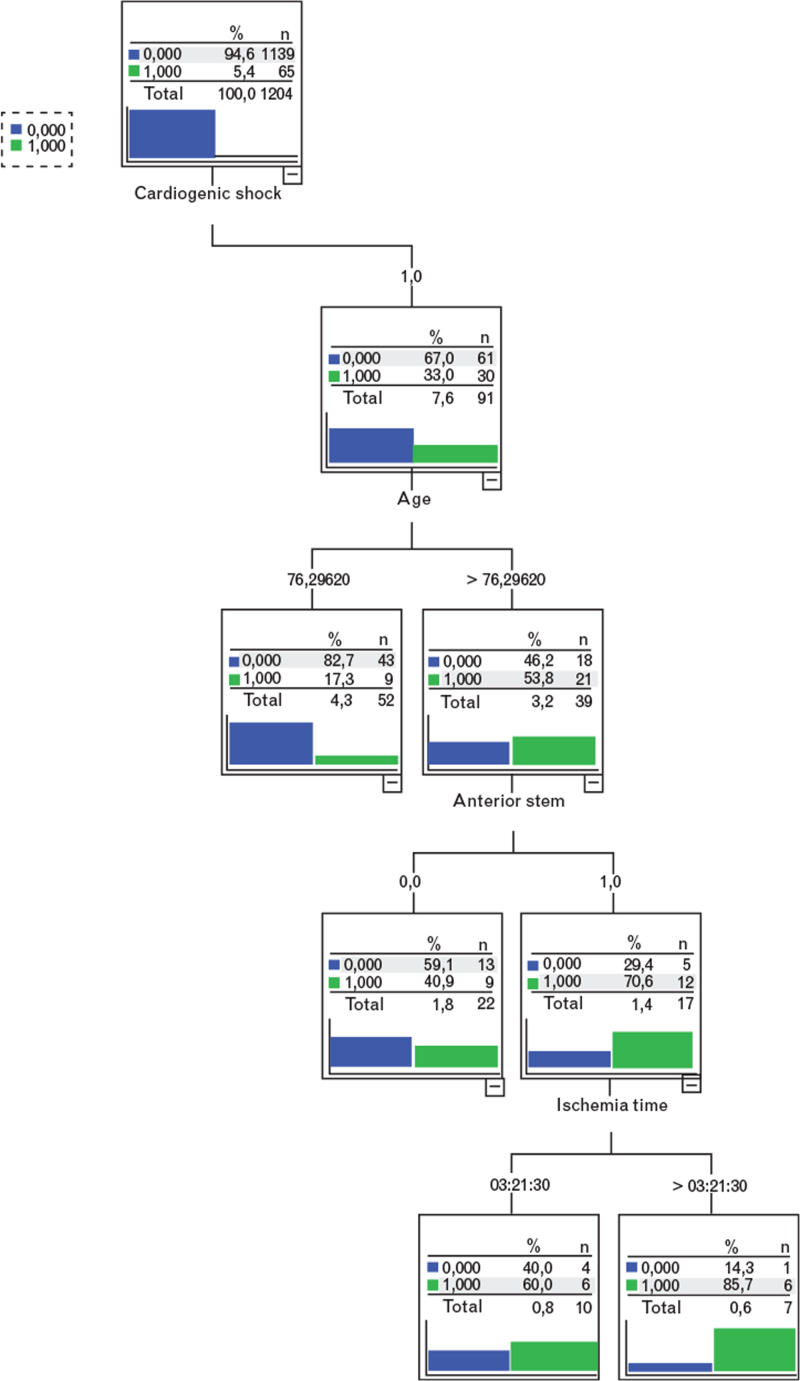
30-day mortality decision tree in the study population according to cardiogenic shock, age, anterior STEMI and ischemia time. 1/Green = % of patients who died within 30 days.

At multivariate analysis, the variables significantly associated with 30-day mortality in the CS population were age (OR 1.246; 95% CI 1.045–1.141; *P* = 0.003), final TIMI flow 2 or 3 (OR 0.058; 95% CI 0.004–0.785; *P* = 0.032) and ischemia time (OR = 1.269; 95% CI 1.001–1.609; *P* = 0.049) (Table 4). Finally, a decision tree (Fig. 2) showed that CS patients above 76 years of age, with an anterior STEMI and an expected ischemia time above 3 h and 21 min had the highest 30-day mortality (85.7%).

**Table 4:** Univariate and multivariate analysis of 30 days mortality in patients with STEMI and cardiogenic shock

Univariate analysis				Multivariate analysis			
Variable	OR	CI 95%	*P*-value	Variable	OR	CI 95%	*P*-value
Age	1.06	1.02–1.11	0.005	Age	1.14	1.05–1.25	0.003
Final TIMI flow 2–3	0.08	0.01–0.35	0.002	Final TIMI flow 2–3	0.06	0.004–0.785	0.032
Ischemia time	1.11	0.97–1.29	0.130	Ischemia time	1.27	1.001–1.609	0.049
Anterior IMA	2.52	1.03–6.39	0.046				
CKD	3.90	1.16–15.6	0.036				

### Age and mortality

**Fig. 3:**
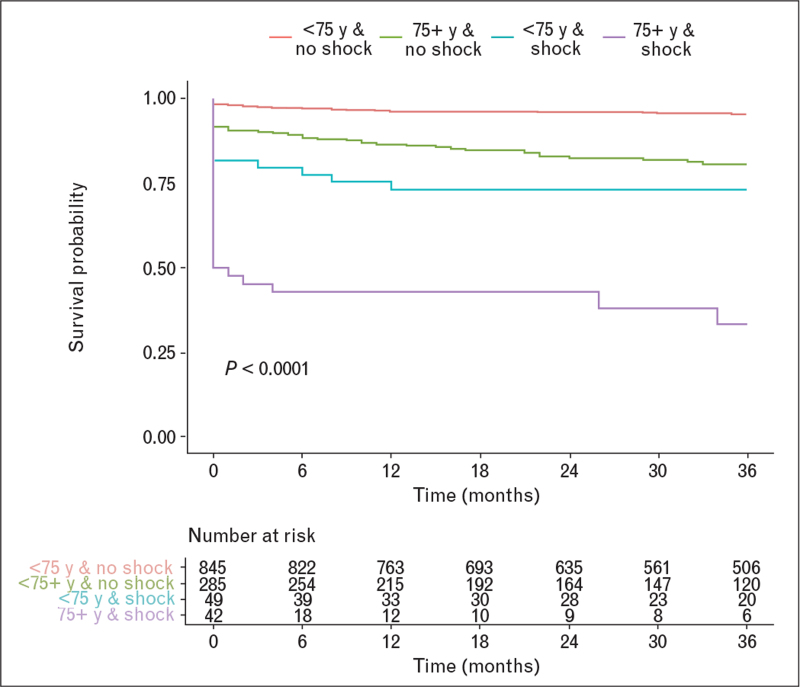
Kaplan–Meier survival analysis according to age and cardiogenic shock status. Red line: patients younger than 75 years old and no shock; green line: 75 years old and older and no shock; blue line: younger than 75 years old and shock; purple line: 75 years old and older and shock (*P* < 0.001).

In Fig. 3, we reported Kaplan–Meier survival curves according to age and CS status.

Interestingly no-CS patients below the age of 75 year have only 1.1% in-hospital mortality whereas those who are 75 years old or older have 8.3% in-hospital mortality. CS further increases in-hospital mortality in patients below 75 years of age (18.4% in-hospital mortality) and profoundly affected prognosis in elderly patients with 47.6% of in-hospital mortality suggesting a synergistic effect of age and CS presentation on prognosis.

## Discussion

In an all-comers contemporary series of real-world STEMI patients, the principal findings of our study that focused on CS patients are: (1) a nonnegligible number of STEMI patients (7.5%) presented with CS; (2) we confirmed that the incidence of CS increases with age,^8,15^ which is a major independent predictor of mortality; (3) successful PCI and short total time of ischemia are factors which have impact on prognosis, highlighting the importance of STEMI network organization, and application of current reperfusion techniques; (4) a subgroup of elderly patients with CS presentation due to anterior STEMI and with long ischemia time presented a severe prognosis despite revascularization.

Mortality rate after STEMI has decreased over the past two decades,^16,17,18^ and we confirmed very good outcomes especially in young STEMI patients without CS (1.1% in-hospital mortality). Conversely, STEMI presenting with CS have still high 30-day mortality (33%). As expected, CS patients compared with no-CS patients were older, had more often intercurrent cardiac arrest and their renal function was worse compared with no-CS patients, probably reflecting both a more severe comorbidity profile before the event, and the results of severe end-organ hypoperfusion. Interestingly we have found an increased presence of female patients in the CS setting compared with the no-CS setting. Women are on average older than men, they have a worse risk profile (more hypertension, diabetes mellitus, peripheral vascular disease), are more prone to developing mechanical complications and they have more often microvascular dysfunction.^19^

Ischemia time was shorter in CS patients compared with no-CS patients, probably because of shorter ‘patient delay’, an important component of ischemia time,^20^ due to the more severe symptoms. Indeed, the presence of higher peak troponin and lower LVEF indicates more extensive damage. In addition, CS patients have more often multivessel coronary artery disease.

Our registry included a high rate of right ventricular MI in the CS population (1/4 of CS patients, and 4% of the total registry). This may explain also the better LVEF and the lower heart rate (due to bradyarrhythmia during the acute phase) compared with other major studies.^7,21^ Importantly RV dysfunction in STEMI has been shown to be an important predictor of mortality.^22^ However, patients with transient RV dysfunction have a better prognosis compared with patients who have persistent RV dysfunction after revascularization.^22^

Strategies of revascularization in CS have been recently evaluated by the CULPRIT-SHOCK^7,12^ trial. Complete revascularization in multivessel patients undergoing pPCI was found to be associated with worse outcome (a composite of mortality and need for renal replacement therapy), and therefore in patients with CS multivessel PCI during pPCI it is no longer recommended. Usual practice in our center is to not perform the nonculprit lesions during the index procedure of STEMI presentation but to defer the procedure.^23^ Thus all patients with CS and multivessel disease were treated with PCI of the culprit lesion only.

More evidence regarding the potential role of mechanical circulatory supports [Impella, TandemHeart, extracorporeal membrane oxygenation (ECMO), etc.] in CS patients is necessary also considering the downgrading of the intra-aortic balloon pump (IABP) recommendation in the guidelines.^24^ Indeed IABP failed to show a significant benefit on 30-day mortality in patients with CS complicating acute myocardial infarction.^13^ In our population IABP was applied in 30.3% of CS patients, which is a fairly high percentage if compared with that reported (7%) in the recent CULPRIT SHOCK trial.^7^ However, our registry included patients treated before the IABP use was downgraded by guideline in the CS setting. To date, Impella did not show superiority in terms of short-term mortality against IABP in several different trials,^25,26,27^ whereas there are some favorable data in support of ECMO.^28^ These devices, however, are mainly reserved for young patients. In this regard, age was found as a major factor influencing prognosis. As can be observed in Fig. 3, the stratification according to age (cutoff 75 years old) and the presence or absence of CS is itself capable of affecting heavily the prognosis: patients younger than 75 years old and without CS have an excellent prognosis whereas patients with the combination of age>75 years and CS presentation have a dramatic increase in 30-day mortality of up to 50%. Elderly patients have various reasons to have a high mortality: the aging process is associated with frailty and higher prevalence of comorbidities;^29^ moreover elderly patients have often atypical symptoms, which may delay prompt diagnosis. However, a decline in mortality of elderly CS patients has been observed in the last few years, possibly because of more extensive use of PCI in these patients.^30^ Even the data from the SHOCK trial confirmed the advantage of PCI in elderly patients.^21^ As shown in our registry, an effective revascularization in CS patients is paramount, indeed obtaining a final TIMI flow of 2 or 3 was an independent prognostic factor associated with lower 30-day mortality. Despite current STEMI guidelines^24^ not reporting specific distinctions on the management of elderly patients, recent data have indicated that PCI is increasingly performed in patients older than 75 years; however, 57.8% of elderly patients with CS are not treated invasively.^31^ This is probably due to the presence of multiple chronic conditions, worse disease burden, and limited life expectancy as assessed by interventional cardiologists.^32^ A decision tree based on the parameters readily available at the moment of the FMC (CS presentation, infarction site, age and total ischemia time) showed that anterior STEMI patients older than 76 years with an ischemia time exceeding 3 h 21 min had 85.7% mortality at 30 days representing patients at highest mortality risk. Although we have identified patients who have the lowest probability of survival despite revascularization, primary PCI remains the best possible treatment for CS patients; however, due to the observational nature of our study we cannot drawn any conclusion on the impact of survival of primary PCI in these extremely high-risk shocked patients.

### Limitations

We must acknowledge limitations to our study. First, the results of our findings should be interpreted in light of the common limitations of a registry-based cohort study. Second, the population is relatively small, in particular when subgroups are taken into account. We did not evaluate the role of acute kidney injury during hospitalization on outcomes. Finally, our pPCI registry included patients considered amenable to pPCI. Nevertheless, some of these limitations are also the strength of these data which provide a pure representation of real-world all-comer STEMI patients treated with pPCI.

## Conclusion

In CS patients, age is a relevant negative factor whereas a successful PCI is positively correlated with survival, confirming that prompt revascularization is pivotal in the management of STEMI complicated by CS. However, a subgroup of elderly patients with CS presentation due to anterior STEMI and with long ischemia time presented a severe prognosis despite revascularization. Whether pPCI may have an impact on survival in a very limited number of irreversibly critically ill patients remains uncertain and the identification of these irreversibly shocked patients is nowadays challenging.

## Acknowledgements

Thanks to all the operators of the STEMI network, including the territorial and hospital emergency services of Friuli Venezia Giulia, for their competent and passionate daily work.

### Conflicts of interest

There are no conflicts of interest.
